# Human Movement Recognition Based on the Stochastic Characterisation of Acceleration Data

**DOI:** 10.3390/s16091464

**Published:** 2016-09-09

**Authors:** Mario Munoz-Organero, Ahmad Lotfi

**Affiliations:** 1 Telematics Engineering Department, Universidad Carlos III de Madrid, Avda de la Universidad, 30, E-28911 Leganés, Madrid, Spain; 2 School of Science and Technology, Nottingham Trent University, Nottingham NG11 8NS, UK; ahmad.lotfi@ntu.ac.uk

**Keywords:** human movement detection, activities, wearable sensors, fall detection

## Abstract

Human activity recognition algorithms based on information obtained from wearable sensors are successfully applied in detecting many basic activities. Identified activities with time-stationary features are characterised inside a predefined temporal window by using different machine learning algorithms on extracted features from the measured data. Better accuracy, precision and recall levels could be achieved by combining the information from different sensors. However, detecting short and sporadic human movements, gestures and actions is still a challenging task. In this paper, a novel algorithm to detect human basic movements from wearable measured data is proposed and evaluated. The proposed algorithm is designed to minimise computational requirements while achieving acceptable accuracy levels based on characterising some particular points in the temporal series obtained from a single sensor. The underlying idea is that this algorithm would be implemented in the sensor device in order to pre-process the sensed data stream before sending the information to a central point combining the information from different sensors to improve accuracy levels. Intra- and inter-person validation is used for two particular cases: single step detection and fall detection and classification using a single tri-axial accelerometer. Relevant results for the above cases and pertinent conclusions are also presented.

## 1. Introduction

Human Activity Recognition (HAR) systems are already integrated into many of our daily routine activities [1]. Applications, such as Google Fit [2] or Apple Health [3], are able to detect some activities, such as walking and running, that are linked to health and fitness parameters. Many other HAR-related applications are also available either using the sensors embedded in smart phones or using wearable devices. Applications such as Lumo [4] are developed to provide a gentle vibration when a person slouches to remind them to sit or stand straight and correct their posture [5]. In fact, HAR algorithms based on applying machine learning techniques to data gathered from wearable devices [6] have established themselves as a convenient alternative for vision-based activity detection algorithms [7,8]. Using wearable sensors provides a non-intrusive, always available companion compared to vision-based systems [6].

Health monitoring systems based on recognising human activities from wearable devices are applied to remote health monitoring for long-term recording, management and clinical access to patient’s activity information [9,10]. Knowing the activities that a patient with long-term conditions, such as Cardio Vascular Disease (CVD), Chronic Obstructive Pulmonary Disease (COPD), Parkinson’s Disease (PD) or diabetes, is performing could help in providing assistance [9]. In fact, the activity performed is part of the environment of a particular user, which provides valuable information for the implementation of personal recommender systems [11]. Home monitoring, assisted living and sports and leisure are other applications that are benefiting from wearable sensors to detect human activities [12].

Although detecting activities provides relevant contextual information for many applications, sometimes, it is desirable to go further in order to detect specific movements and gestures (either those made inside activities [13] or those that occur in sporadic moments [14]). Spotting sporadically-occurring movements from continuous data streams is a challenging task, especially when pre-detection techniques are to be deployed on the sensor systems themselves. Wearable sensor devices are relatively limited with their computational performance. In addition, battery consumption, as well as memory usage are other limitations [6]. Having to recharge the batteries of wearable sensors too often is a major drawback for their adoption by many users. Communications over wireless interfaces should therefore be minimised as much as possible. This will require performing pre-detection computations on wearable sensor devices. However, extensive computations on limited devices are also linked with energy consumption, and therefore, simple, but reliable algorithms should be conceived of for pre-detecting segments in the data stream from each sensor in order to limit communications to those particular segments [14]. The overall performance of an HAR system in terms of accuracy, precision and recall could later be improved by combining the information from several sensors [11].

This paper proposes a novel approach to in-sensor pre-detection of movements using an algorithm that concentrates on the stochastic properties of local maxima and minima from the sensed data stream in order to detect specific movements. The hypothesis behind the design of the algorithm is that some atomic movements can be detected with sufficient accuracy. This can be achieved by considering the stochastic properties of the values and time variations of local maximum and minimum points from the sensed data (points that are easy to detect from raw sensed data by using simple mathematical operations and only requiring a limited amount of memory, adapted to low energy consumption and computational requirements when implemented on sensor devices). The algorithm could be trained for a particular individual or partially independent of users by considering data from different users when training the algorithm. The performance of the pre-detection of movements could be improved if several movements are performed together within a gesture. Combining movements in order to detect gestures is also a challenging task in general due to the variability of execution (and their related time series) both for intra- and inter-person data. Some approaches are based on requiring non-overlapping movements with pauses between consecutive movements [15]. Moreover, using data from wearable sensors introduces additional challenges, such as noise and overlapping movements from the sensor itself if not tight to the body.

This paper will also present the results for two particular scenarios in which the proposed algorithm is implemented in order to (a) detect single steps and (b) identify and classify different types of falls using a single tri-axial accelerometer. The single step detection algorithm is trained to detect the behaviour when each foot initially comes in contact with the ground. By combining consecutive movements of “foot-ground contact”, a walking gesture can be detected. Moreover, sporadic failures in the detection of a particular step when walking could be used to fine tune the detection parameters of the algorithm by incorporating the missing steps into a new training phase of the parameters for a particular person. The fall detection and classification algorithms are able to detect and categorise the type of fall out of 30 falls in a public database.

The rest of the paper is organised as follows. Section 2 presents an overview of the previous work and justifies the research in this paper. Section 3 details the proposed algorithm. Section 4 particularises the algorithm for the particular case of step detection. Section 5 presents the adaptation of the algorithm for detecting and classifying falls. Section 6 presents the details of the experiment carried out to validate the algorithm. Section 7 captures the conclusions and the future work.

## 2. Related Work

The first papers on HAR were published in the late 1990s [16]. There are two main approaches to implement HAR systems, using external and wearable sensors [6]. In the former, the devices are fixed at predefined locations of observation in order to monitor the user (using cameras or microphones, for example). In the latter, the devices are attached (worn) by the user. Each approach has its advantages and disadvantages. However, some privacy, as well as availability issues are promoting a shift in research towards wearable sensors.

The availability of sensors, such as Microsoft Kinect [17], PrimeSense Carmine and Leap Motion [18], have helped with the advances in capturing human motion. The non-intrusive nature of sensors, their low cost and wide availability for developers have inspired numerous healthcare-related research projects in areas, such as medical disorder diagnosis [19], assisted living [20] and rehabilitation [21]. However, most of these tools were originally developed for computer games.

In this paper, the concentration will be on wearable sensors to solve human movement detection problems. To detect activities with wearable sensors, two major approaches are adopted by researchers. The reviewed literature is presented below.

### 2.1. Sliding Window-Based Approaches

The data stream is divided into time overlapping windows to detect a particular activity among a set of pre-trained activities. The size of the window is defined so that enough information about the activity is present and only one activity is performed in that window (stationary). The window length is related to the achieved performance [22]. In fact, most of the detection errors occur in windows that contain transitions from one activity into a different activity. The data stream is pre-processed into some pre-selected features. Two major sets of features could be extracted from the time series data; statistical and structural [23]. In order to minimise the impact of the set of selected features, sparse representations could be used using the training samples directly as the basis to construct an over-complete dictionary [24]. The application of different classification algorithms to the detected features, such as decision trees, Bayesian methods, Hidden Markov Models (HMM), nearest neighbours, Support Vector Machines (SVM) or ensembles of classifiers, could determine the activity being performed with accuracies greater than 95% if simple activities are being classified [6,25,26]. Taking into account the uncertainty and flexibility in human activities, fuzzy rule-based systems [27] or other forms of fuzzy classifiers [28] are used and show their effectiveness in classifying activities.

The performance of existing HAR approaches based on inertial sensors for detecting activities and transitions between different activities and postures is affected by sensor placement and relative to body movements. To overcome these limitations, one possibility is to use additional information or sensor drift compensation techniques. The authors in [27] propose to use the additional information from a barometric pressure sensor to improve the performance of a tri-axial accelerometer. The presented research in [29] proposes the use of a combination of sensors to compensate for the weaknesses of either sensor in recognising various activities. In fact, to be able to classify more complex activities, such as taking a medicine or cooking, it could be essential to combine the information gathered from several sensors [30]. In [31], the authors propose a mechanism to compensate the accelerometer bias by computing the average of each acceleration component over the sliding window. A similar approach of using a low-band averaging filter to detect sensor relative movements with the body is used in this paper. The windowed average of each acceleration component is used to estimate the orientation of the sensor so that using rotation operations, vertical and longitudinal acceleration components could be extracted.

### 2.2. Decomposition of Activities into Basic Primitives

To decompose the activities into a sequence of elementary building blocks, two major approaches are adopted. A top-bottom approach first tries to identify the activity that a user is performing and then divides the activity into smaller segments containing specific movements inside the activity. A bottom-up approach focuses on detecting movements and tries to compose them into gestures and activities. The authors in [32] propose a motion primitive-based model that captures the invariance aspects of the local features and provides insights for better understanding of human motion. The continuous activity signal is transformed into a string of symbols where each symbol represents a primitive. String-matching-based approaches can then be used to detect activities out of detected primitives or other methods, such as “a bag of features” could be used to improve the detection performance [32]. The authors in [13] also approach the problem of activity recognition by modelling it as a combination of movements. They use a two-step approach in which they first use a sliding window to detect activities and later try to classify movements inside activities. The authors in [14] focus on directly detecting sporadic movements by directly processing the time series of the data sensed. They use a modified version of the Piecewise Linear Representation (PLR) algorithm in order to detect segments in the data stream that may contain movements of interest. They use different sensors that are combined in order to improve the detection accuracy. The approach is promising, but the algorithm deployed on sensor devices could be better optimised in order to reduce the complexity of the PLR algorithm, as well as to take into account the stochastic characteristics of the detected points of interest (linear pieces). The authors in [33] also decompose activities into atomic pieces and propose the use of shapelets as the basis to classify atomic activities. Therefore, human movements are recognised by calculating the distance between the sensed time series and a dictionary of pre-recorded segments, which try to capture the particularities of each movement. They propose the use of the Euclidean distance, although recognise that other alternatives would also be applicable. Once atomic activities are recognised, they use atomic activities to assess sequential, concurrent and complex activities. Although the shapelet approach tries to simplify the information in the time series recorded from wearable sensors into significant fragments, the variations in intra- and inter-person execution could be big, and a measure based on the distance with pre-recorded segments will not take into account the variety of cases unless a complex and high volume training process is performed.

Taking into account the low energy constrains for on-sensor implementation, the approach presented in this paper is to generate a simple movement detection algorithm. This is based on a stochastic model characterising the local maxima and minima relationships from the time series measured from a single tri-axial accelerometer. The stochastic model will capture the information from the execution of a particular movement by different people and different execution speeds. Movement elasticity based on previous proposals, such as Dynamic Time Warping (DTW) [34], do not properly consider non-linearities and do not provide the required flexibility for capturing rich human movements. The time elasticity model proposed in this paper will be based on the characterisation of the stochastic features of inter-time and amplitude variations among local maxima and minima when performing the same movement by different people at different speeds. The average gravity estimation and compensation method presented in [31] will be used to minimize the impact of sensor placement and relative to the body over the time sensor movements. Estimating the gravity vector allows us to apply rotation operations to isolate vertical and longitudinal movement components.

## 3. Atomic Movement Detection Algorithm

**Figure 1:**
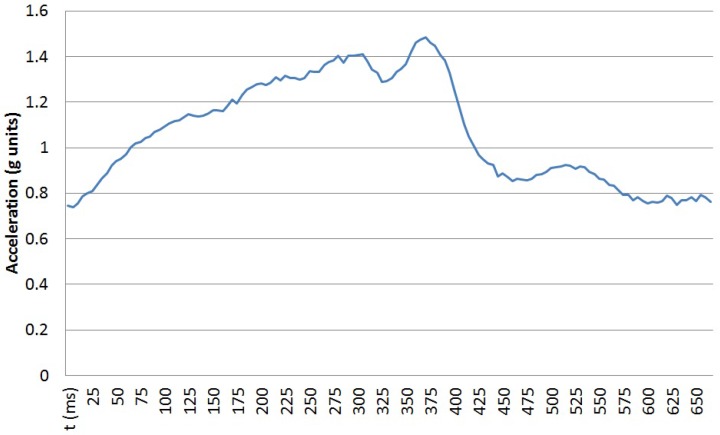
Vertical acceleration in a single step.

**Figure 2:**
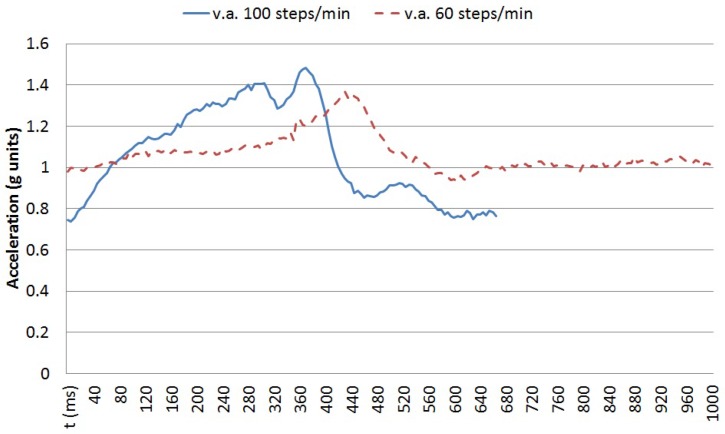
Vertical acceleration in a single step at different speeds.

Atomic movements are short gestures or primitives executed when performing more complex activities or in sporadic ways. Walking a step or moving a spoon to one’s mouth are two simple examples. Each atomic movement can be performed at different speeds and following different trajectories by different people or even by the same person at different moments. The traces of the execution of each atomic movement can be captured by wearable sensors, such as tri-axial accelerometers. These sensors capture data as time series of scalar values. The vertical acceleration for a single step is shown in Figure 1. Executing the same movement at different speeds does not provide a time-scaled version of the sensed data, but a non-linear variation over time. Figure 2 captures the vertical acceleration for a single step at two speeds for the same person: 60 steps per minute and 100 steps per minute. In order to detect the execution of a particular movement, an algorithm based on the stochastic characterisation of the movement performed at different speeds by different users is proposed. Using a tri-axial accelerometer, the temporal elasticity of the movement is described by the changes both in amplitudes and time shifts among the different axes.

Let us call $\mu_{i}$ the speed of executing a particular instance *i* of a particular movement. Let us call $\delta_{i}$ the duration of the execution of that movement and call $\delta_{max}=\forall_{i}^{max}\delta_{i}$. For each segment of the sensed data of $\delta_{max}$ duration, from which it is desired to estimate the speed of execution, the posterior distribution for the speed of execution of the atomic movement over the sensed data is given by Equation (1).

(1) $$p\left(\mu_{i}|D\right)=\frac{p\left(D|\mu_{i}\right)p\left(\mu_{i}\right)}{p(D)}$$

In the above equation, *D* represents the data extracted features in a particular segment. The probability of the data based on a particular speed of execution $p(D|\mu_{i})$ could be estimated by extracting the stochastic properties from sample data by different users. In order to use Equation (1), data could be described using different features as described in Section 2. Our approach is to characterise the time series using features that can be detected with low energy pre-processing algorithms. In particular, it is proposed to characterise the sensed time series by the times and amplitudes of the local maximum and minimum in a $\delta_{max}$ time window. When using tri-axial accelerometers, different sub-features can be computed in order to capture the particularities of each movement, such as the time difference between the maximum value of the vertical acceleration and the horizontal acceleration or the relative variation in amplitude between vertical and horizontal accelerations. For each feature, a Gaussian mixture model will be used in order to simplify the stochastic representation and the required training phase. The probability mass of a particular feature extracted from the data samples $D_{k}$ executed at speed $\mu_{i}$ could be approximated by Equation (2):

(2) $$p\left(D_{k}|\mu_{i}\right)\approx N\left(\lambda_{ki},\sigma_{ki}\right)$$

where *N* is the normal distribution with *λ* mean and *σ* variance. In order to simplify the model, a combined multivariate Gaussian probability for all features could be approximated by assuming independence as captured in Equation (3).

(3) $$p\left(D|\mu \right)\approx \prod_{k}p\left(D_{k}|\mu_{i}\right)$$

The prior distribution over $\mu_{i}$ could capture the prior knowledge of the particularities when performing the particular movement by a particular individual. In the case of step detection for example, the normal walking speed for a particular user could be used to generate a user-fitted prior distribution.

## 4. Atomic Movement Detection Algorithm for Step Detection

In order to validate the proposed approach, following the design criteria presented in Section 3, a new algorithm is proposed here. This algorithm is used for the particular case of step detection.

The generic problem of detecting specific movements (or patterns) from sensor-generated time series can be approximated in two phases: feature extraction and movement classification based on the extracted features [23]. Both phases could be implemented in the sensor device and/or on external computing systems. However, the final architecture should provide energy efficiency awareness in order to maximise the battery lifetime for sensors and therefore maximise their user acceptance. Deploying on-sensor algorithms will minimise communication energy costs at the expense of energy expended on embedded computations. On the other side, sending raw data to external systems will increase the communication energy requirements when trying to minimise sensor energy consumption on computations.

Our aim is to optimise energy requirements by performing on sensor pre-detection based on low energy features in order to minimise both computing and communication requirements from the energy perspective. Complex features based on time-frequency or wavelet transforms [6] or full time-series computations based on approximated shapes [14,23] and distance-based similarities will penalise the energy required for on-sensor device computations. Approaches based on sparse dictionaries [24] will require sending the complete sensed data to external servers, increasing the communication energy costs. In order to simplify the feature extraction from the acceleration time series, the proposed algorithm only uses the amplitudes and times of consecutive maxima and minima data points. A stochastic model for estimating the likelihood of a particular data segment to be a step based on the times and amplitudes of consecutive maxima and minima is developed and trained so that the operations required for detection are energy efficient. In particular, the algorithm proposed in this section has selected two simple features, which have shown good correlations with minimal complexity for step detection. The selected features are:

(4) $$\begin{array}{c} f_{1}=A\left[Max\left(G_{z}\right)-Min\left(G_{z}\right)\right] \end{array}$$

(5) $$\begin{array}{c} f_{2}=T\left[Max\left(G_{y}\right)-Min\left(G_{y}\right)\right] \end{array}$$

where *A* refers to the amplitude of the sensed time series, $G_{z}$ is the vertical acceleration, $G_{y}$ is the acceleration in the movement direction, *T* is the time difference and Max and Min represent the local maximum and minimum in the time window.

Assuming that features are independent of each other, at first, the speed of the execution of the movement is estimated by using the following equation:

(6) $$p\left(\mu_{i}|f_{1},f_{2}\right)=\frac{p\left(f_{1}|\mu_{i}\right)p\left(f_{2}|\mu_{i}\right)p\left(\mu_{i}\right)}{\sum_{i}p\left(f_{1}|\mu_{i}\right)p\left(f_{2}|\mu_{i}\right)p\left(\mu_{i}\right)}$$

In order to apply Equation (6) to a dataset that includes segments of data containing steps and control segments in which no steps are executed (null class), the stochastic information for the null class has also to be taken into account. In our case, the null class is modelled as two single side distributions with an exponential shape as represented in Equation (7):

(7) $$p\left(f_{j}|\mu_{0}\right)=\frac{1}{2}\lambda_{01}e^{-\lambda_{01}\left(f_{j}-\mu_{01}\right)}+\lambda_{02}e^{-\lambda_{02}\left(\mu_{02}-f_{j}\right)}$$

where $\mu_{0}$ represents the null class and $\lambda_{01}$, $\mu_{01}$, $\lambda_{02}$ and $\mu_{02}$ are the parameters of the distribution.

After estimating the values for $p(\mu_{i}|f_{1},f_{2})$ based on the training data, these values are used to assign a probability for a segment of validation data to contain a step using Equation (8).

(8) $$p\left(step|f_{1},f_{2}\right)=\sum_{i}p\left(step|f_{2},f_{1},\mu_{i}\right)p\left(\mu_{i}|f_{1},f_{2}\right)=\sum_{i>0}p\left(\mu_{i}|f_{1},f_{2}\right)$$

The output of Equation (8) will provide a measure of likelihood that a step movement is performed. In order to simplify the proposed model, the $\mu_{i}$ variable is sampled at particular points of interest that capture the range of walking speeds that will be detected. In our case, the stochastic distributions of $f_{1}$ and $f_{2}$ are characterised based on vales for $\mu_{i}$ in the range of 60 to 100 steps per minute (corresponding to walking speeds between 3 and 5 km/s approximately). Equation (6) is approximated by a Gaussian mixture model based on normal distributions for the selected values for $\mu_{i}$. In this model, computing Equation (8) will be based on simple evaluations of the normal distribution based on the values of $f_{1}$, $f_{2}$ and the result of Equation (6).

In order to compensate the misplacement of the sensor device (a common issue with wearable sensors for daily activity monitoring) a low-band filter based on the averaging over a 2-s window is implemented (as proposed in [31]). This moving average is able to detect both the original placement rotations of the sensor, as well as slow drifts in the sensor location relative to the human body. The average vector detects the gravity component. The projection of each accelerometer sample with the average vector is used to estimate the vertical acceleration component in our algorithm.

## 5. Atomic Movement Detection Algorithm for Detecting and Classifying Falls

In order to validate the proposed approach, following the design criteria presented in Section 3, a second algorithm is proposed in this section. This algorithm is used for the particular case of fall detection and classification.

The proposed algorithm is divided into two consecutive phases: fall detection and fall classification. In order to detect falls, the algorithm proposed in this section has selected two simple features, which have shown good correlations with minimal complexity for fall detection:

(9) $$\begin{array}{c} \;f_{1}=A\left[Max\left(G\right)-Min\left(G\right)\right] \end{array}$$

(10) $$\begin{array}{c} f_{2}=T\left[Min\left(G\right)-Max\left(G\right)-Min\left(G\right)\right] \end{array}$$

where *A* refers to the amplitude of the sensed time series, *G* is the amplitude of the acceleration vector, *T* is the time difference and Max and Min represent the local maximum and minimum in the time window.

In order to detect falls, the probability of having a fall conditioned to having measured $f_{1}$ and $f_{2}$ should exceed a certain threshold, *τ* as captured in Equation (11).

(11) $$p\left(fall|f_{1},f_{2}\right)>\tau_{1}$$

Assuming that $f_{1}$ and $f_{2}$ are independent variables, a fall is detected when the condition in Equation (12) is met.

(12) $$p\left(f_{1}|fall\right)p\left(f_{2}|fall\right)>\tau_{2}$$

Based on the results in this study, a value of 0.1 for $\tau_{2}$ has proven to be valid to distinguish 100% of falls in the database [35].

Once a fall is detected, the proposed algorithm will estimate the class of fall that occurred. Based on the available experimental data, the attention is focused on detecting frontal falls. Falls have occurred when the user is walking or sitting on a chair. The database in [35] contains 30 falls; six of them are frontal falls with no rolling over once the user contacts the ground. Out of these six falls, three are falls starting from a walking state and three falls start from a sitting position. The following three basic features are used:

(13) $$\begin{array}{ccc} f_{1} & = & A[G_{x}@Max\left(G\right)] \end{array}$$

(14) $$\begin{array}{ccc} f_{2} & = & A[G_{y}@Max\left(G\right)] \end{array}$$

(15) $$\begin{array}{ccc} f_{3} & = & A[G_{z}@Max\left(G\right)] \end{array}$$

where *A* refers to the amplitude of the sensed time series, *G* is the amplitude of the acceleration vector and $G_{x}$, $G_{y}$ and $G_{z}$ are the individual acceleration components.

All available 30 falls in the database are classified into three classes:

(16) $$\begin{array}{ccc} c_{1} & = & \text{frontal fall when walking} \end{array}$$

(17) $$\begin{array}{ccc} c_{2} & = & \text{frontal fall when sitting } \end{array}$$

(18) $$\begin{array}{ccc} c_{3} & = & \text{Other fall } \end{array}$$

The detection of each class implies having the probability for the class conditioned to the measured feature above a certain threshold. The computation can be performed based on Equation (19).

(19) $$p\left(f_{1}|c_{i}\right)p\left(f_{2}|c_{i}\right)p\left(f_{3}|c_{i}\right)>\tau_{i}$$

## 6. Results Validation

This section presents the validation of the results for detecting steps and falls based on the algorithms proposed in the previous sections. We use the information in two public datasets in order to validate the results. The dataset in [36] is used for step detection, and the dataset in [35] is used to detect and classify falls.

### 6.1. Step Detection

**Figure 3:**
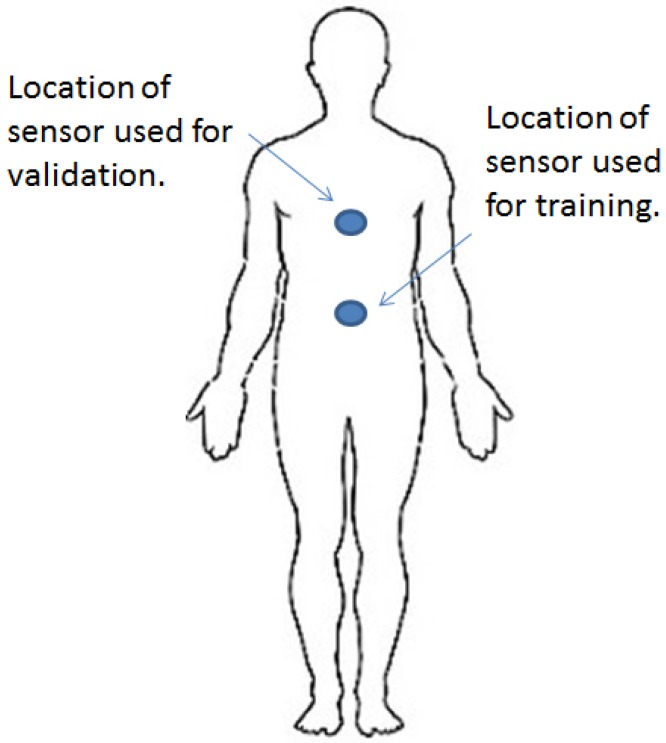
Location of sensors for training and validation.

In order to assess the inter-person validity, a different set of users and hardware devices is used for training and validation. An Android Nexus 6 device is fixed to the body close to the abdomen for training. For validation, the chest-located accelerometer data available from dataset [36] are used. Figure 3 illustrates the locations of the sensor devices for training and validation. In both cases, torso-attached sensors are used to get similar time series. Based on the bio-mechanics of walking, the acceleration of the upper and the lower torso may be slightly different in amplitude. A compensation method is implemented to correct these differences. Using the Android Nexus 6 device placed at both upper and lower torso locations, several measurements, while walking at the same speeds, are taken and analysed. Table 1 captures the differences in the amplitude signals for different axes at both locations. Different hardware from different manufacturers at different sample rates and slightly different sensor locations, as well as a different set of users for training and validation are used in order to assess the generalisation of the results independently of the user, the sensor device and the exact placement of the sensor. To be able to apply the training of the algorithm with our collected data to the data in the dataset in [36], only walking on a hard surface is considered.

The dataset in [36] contains the information for 13 activities performed by 19 participants (each wearing four accelerometer devices in four different parts of the body). In our experiment, the chest accelerometer is used since the data are similar (although not exactly the same) to the training scenario in which the accelerometer is used in a mobile device when maintaining the device close to the participant’s abdomen. The performed 13 activities in the dataset are: sitting, lying, standing, washing dishes, vacuuming, sweeping, walking, ascending stairs, descending stairs, treadmill running, cycling on ergo-meter (50 W), cycling on ergo-meter (100 W) and rope jumping. Our objective is to assess both the sensitivity (the percentage of steps counted when walking) and specificity (the number of false steps detected when not walking).

**Figure 4:**
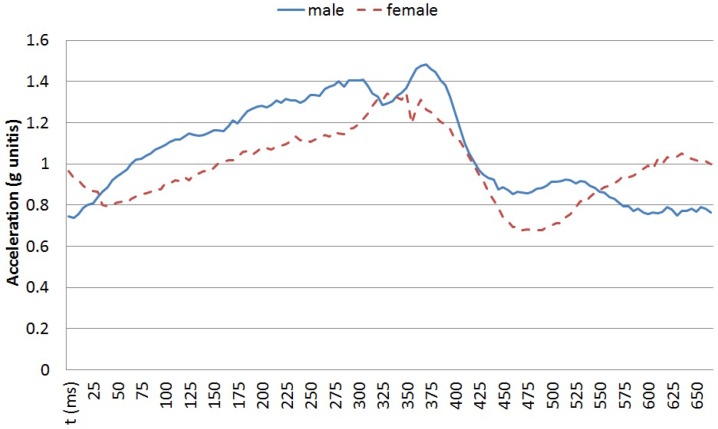
Vertical acceleration in a single step for one male and one female participants at 100 steps per minute.

For the training set, the recordings for the tri-axial accelerometer embedded in an Android Nexus 6 worn close to the participant’s abdomen of three men and three women are used. Each participant walked 20 steps at three different speeds (60, 80 and 100 steps per minute). A metronome was used to guide the participants in keeping the step cadence. The vertical acceleration for a single step for two of the participants walking at 100 steps per second is shown in Figure 4.

**Figure 5:**
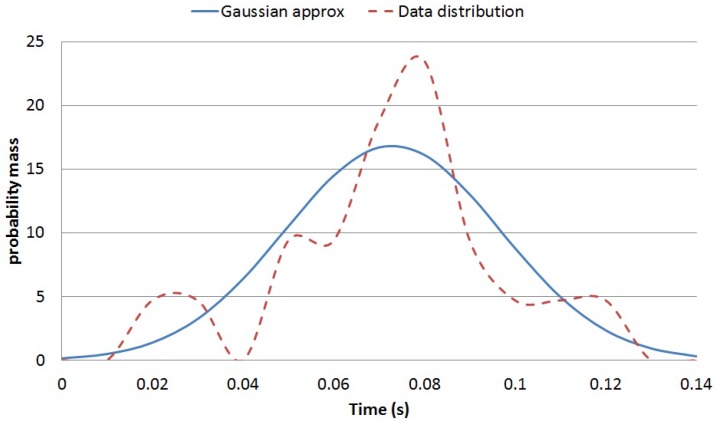
Normal approximation for $f_{2}$ for a participant walking at 100 steps per minute.

Using the data from each participant at each speed, the measured probability mass functions of each feature were approximated using Equation (2). Figure 5 captures the example for Feature 2 for a participant walking at 100 steps per minute. The training results for the conditional distribution $p(f_{1}|\mu_{i})$ and $p(f_{2}|\mu_{i})$ parameters are presented in Table 2 and Table 3, respectively.

**Figure 6:**
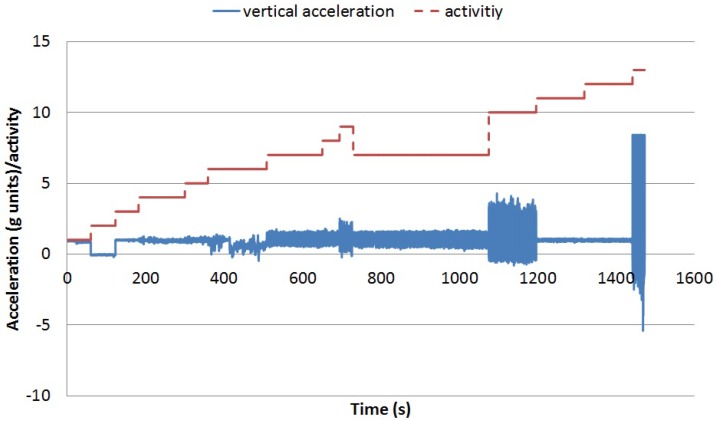
Vertical acceleration over time per activity for Participant 2.

For the validation phase, the dataset in [36] is used. Figure 6 captures the vertical acceleration for one of the participant over time (in seconds) and the activity being performed. Each activity is represented with an associated number according to the following list (from 1 to 13, walking tagged as Number 7):

1. Sitting,
2. Lying,
3. Standing,
4. Washing dishes,
5. Vacuuming,
6. Sweeping,
7. Walking,
8. Ascending stairs,
9. Descending stairs,
10. Treadmill running,
11. Cycling on ergometer (50 W),
12. Cycling on ergometer (100 W),
13. Rope jumping.

**Figure 7:**
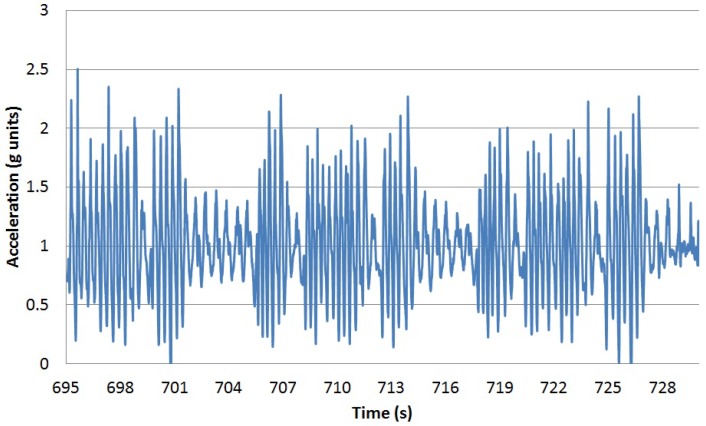
Vertical acceleration over time descending steps for Participant 2.

Apart from the steps performed while walking, there are steps executed inside some of the rest of the activities. Figure 7 illustrates the vertical acceleration for one of the participants when descending stairs. There are three periods in which the participant walks inside the staircase, probably in flat segments connecting segments of stairs.

**Figure 8:**
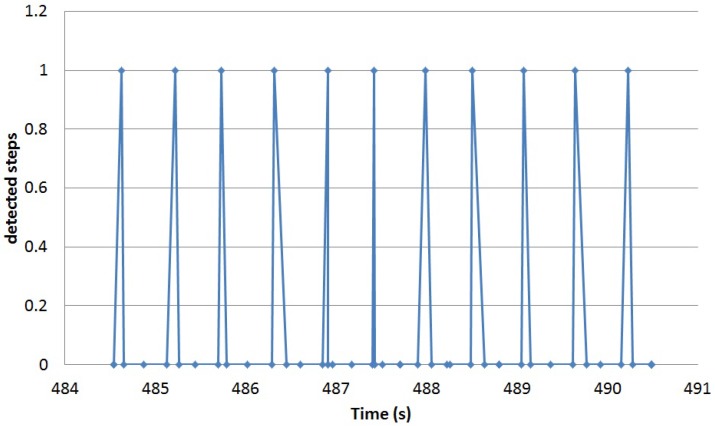
Steps detected in a walking segment.

**Figure 9:**
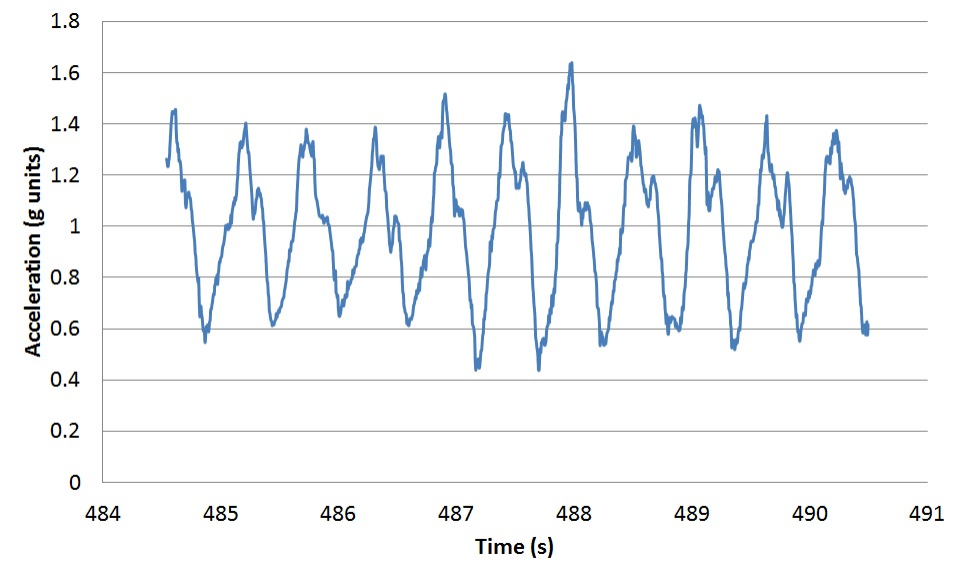
Vertical acceleration for the same segment as in Figure 6.

The results of the algorithm applied to the data while walking are able to detect 91.14% of the steps. Figure 8 shows the moments in time when steps are detected for a segment of the walking data for Participant 1. Figure 9 shows the associated vertical acceleration for the same period of time. The benefits of the proposed approach when applied for the detection of periodic activities (the movement under detection is performed on a periodic base) is that the misclassified movements could be easily post-detected after the detection of two movement events with an inter-time of occurrence around twice the previously detected one. Using this approach to “correct” single misclassified steps in the validation dataset, 99.45% of the steps are estimated. The best classifier in [36] for detecting walking-related segments (KNN) reaches an overall mean classification rate of 97.7%, but without being able to sub-classify walking from ascending steps and running; windows were taken to contain single activities; and four different sensors at four different locations were used at the same time (wrist, hip, ankle and torso).

The proposed algorithm can also be used for detecting the execution of steps on flat surfaces that are performed inside other activities. The algorithm applied to the accelerometer data from the rest of the activities is presented in Table 4. The algorithm is able to detect some of the steps walking on flat segments in activities, such as ascending and descending stairs. In order to validate the performance of the algorithm for the detection of steps in similar activities, the features $f_{1}$ and $f_{2}$ are also calculated for the steps executed when ascending and descending stairs. Both ascending and descending segments of acceleration data contained steps walking on flat surfaces connecting stair fragments. The steps walking on flat surfaces were extracted and labelled as flat surface steps. The confusion matrix is presented in Table 5. All of the steps ascending and descending stairs were classified correctly, and only a small fragment of steps walking on flat segments of the staircase were incorrectly classified as ascending steps.

### 6.2. Fall Detection and Classification

**Figure 10:**
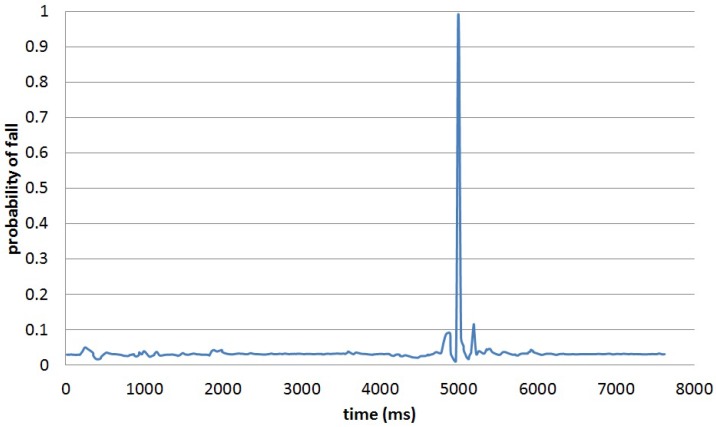
Probability of a fall for Data Sample 9 in the database.

In order to validate the algorithm for detecting and classifying falls, the database in [35] is used. This database contains 30 falls; six of them are frontal falls with no rolling over once on the ground. Out of these six falls, three are falls from a walking state and three falls from a sitting position. Table 6 captures the probability distribution parameters for both $f_{1}$ and $f_{2}$ conditioned to a fall based on the information in the database in [35]. The first feature, $f_{1}$, is designed to capture the sharp accelerations occurring during the impact with the ground, while the second feature, $f_{2}$, is able to validate a fall by considering the time taken for a ground impact in order to distinguish it from other high acceleration movements. Figure 10 captures the result of applying the proposed algorithm for detecting falls to a particular sample in the database. Once a fall is detected, we apply our algorithm for classifying them. Table 7 captures the results of the conditional distributions of each feature for each class of fall as previously defined. The algorithm was able to detect all falls and to accurately classify them in the appropriate class. The major limitation of the database is that all falls are simulated. The authors are currently working to create a database with real falls by monitoring frail people in care homes.

## 7. Conclusions

In this paper, a novel algorithm is presented to detect atomic human movements based on the stochastic properties of local maxima and minima of the sensed time series from a tri-axial accelerometer. The algorithm is designed to work on simple operations, such as storing the maximum and minimum values and times for the three acceleration axes and performing simple estimations for the probability of steps based on the normal distribution. The proposed algorithm is applied to two particular scenarios: detecting single steps while walking and detecting and classifying falls. The training set and the validation set for the step detection have used different individuals, hardware and software for the sensors (with location compensation). The fall classification is tested on a public available falls database.

The results validate that it is possible to detect atomic movements based on the stochastic characterisation of the elasticity of the sensed time series when performing the movement by different individuals at different speeds. The time elasticity is mapped into the deformations in the relative location and values for adjacent maxima and minima.

For future work, our model will be applied for the detection of other atomic movements and the detection of more complex activities by the recognition of a combination of atomic movements. We will also perform an on-sensor deployment to assess real-time detection.

## Figures and Tables

**Table 1:** Differences in sensed data at 60 steps per minute in lower and upper torso locations.

Location	$\sigma_{G_{y}}$	$\sigma_{G_{z}}$
Lower torso	0.0794	0.0830
Upper Torso	0.0848	0.0862

**Table 2:** Training of the algorithm, $f_{1}$ parameters.

*μ*	$\lambda_{f_{1}}$	$\sigma_{f_{1}}$
60 steps/min	0.4846	0.2023
80 steps/min	0.6760	0.2922
100 steps/min	0.8141	0.3581

**Table 3:** Training of the algorithm, $f_{2}$ parameters.

*μ*	$\lambda_{f_{2}}$	$\sigma_{f_{2}}$
60 steps/min	0.1440	0.0826
80 steps/min	0.1188	0.0715
100 steps/min	0.0688	0.0370

**Table 4:** Average number of steps counted per participant for non-walking activities.

Activity	Average Duration (min)	Average Steps Counted
Sitting	1	0
Lying	1	0
Standing	1	0
Washing dishes	2	0
Vacuuming	1	2
Sweeping	1	11
Ascending stairs	<1	18
Descending stairs	<1	7
Treadmill running	2	0
Cycling on ergometer (50 W)	2	0
Cycling on ergometer (100 W)	2	0
Rope jumping	<1	0

**Table 5:** Confusion matrix for detecting steps walking on flat surfaces, ascending stairs and descending stairs.

Classified as	Flat Surface Steps	Ascending Steps	Descending Steps
Flat surface steps	95.9%	4.1%	0%
Ascending steps	0%	100%	0%
Descending steps	0%	0%	100%

**Table 6:** Fall detection algorithm parameters.

Class	$\lambda_{f}$	$\sigma_{f}$
$f_{1}$	7.8549	2.5692
$f_{2}$	136.27	81.36

**Table 7:** Fall classification algorithm parameters.

Class	$\lambda_{f_{1}}$	$\sigma_{f_{1}}$	$\lambda_{f_{2}}$	$\sigma_{f_{2}}$	$\lambda_{f_{3}}$	$\sigma_{f_{3}}$
$c_{1}$	5.2423	2.0798	1.9998	1.0555	−1.8940	0.2080
$c_{2}$	5.1299	2.0878	−0.8582	3.8089	−3.9783	0.1520
$c_{3}$	7.1742	1.4663	1.4240	4.1176	0.0463	3.8780
